# Remote Work, Work Stress, and Work–Life during Pandemic Times: A Latin America Situation

**DOI:** 10.3390/ijerph18137069

**Published:** 2021-07-02

**Authors:** Juan Sandoval-Reyes, Sandra Idrovo-Carlier, Edison Jair Duque-Oliva

**Affiliations:** 1 Facultad de Psicología, Universidad de La Sabana, Chía 250001, Colombia; juan.sandoval1@unisabana.edu.co; 2 INALDE Business School, Universidad de La Sabana, Chía 250001, Colombia; 3 Facultad de Ciencias Económicas, Universidad Nacional de Colombia, Bogotá 111321, Colombia; ejduqueo@unal.edu.co; 4 Universidad Espíritu Santo, Samborondon 104135, Ecuador

**Keywords:** remote work, perceived stress, work–life, COVID-19, Latin America

## Abstract

The COVID-19 pandemic affected the relationship between work and life almost everywhere on the planet. Suddenly, remote work became the mainstream way of working for millions of workers. In this context, we explore how the relationship between remote work, work stress, and work–life developed during pandemic times in a Latin America context. In a sample of 1285 responses collected between April and May 2020, through a PLS-SEM model, we found that remote work in pandemic times increased perceived stress (β = 0.269; *p* < 0.01), reduced work–life balance (β = −0.225; *p* < 0.01) and work satisfaction (β = −0.190; *p* < 0.01), and increased productivity (β = 0.120; *p* < 0.01) and engagement (β = 0.120; *p* < 0.01). We also found a partial moderating effect, competitive and complementary, of perceived stress, and one significant gender difference: when working remotely, perceived stress affects men’s productivity more acutely than women’s productivity.

## 1. Introduction

The coronavirus disease 2019 (COVID-19) pandemic has impacted the work realm, upending the daily routines of millions of people around the globe. Different kinds of measures to control the contagion were put in place by countries’ governments, from quarter long strict lockdowns to mere warnings to avoid crowds. All of them affected work patterns and consequently life routines. The consequences of such alterations vary in degree and quality depending on the detonating stimulus. One such stimulus altering the world of work was remote work or teleworking. 

Starting middle of March 2020, millions around the world began working from home, adjusting their work activities to a different location but maintaining their goals and responsibilities. Latin America was no exception. Countries in this region of the world with fewer health provisions and resources than developed ones adopted similar restrictions, and companies and service providers had to change the way they worked. According to a report from the Economic Commission for Latin America and the Caribbean, one fifth of people in employment have been able to work from home during the pandemic [1] and several countries had to adjust current employment legislation to accommodate for remote working [2]. At the same time, and under these new circumstances, the International Labor Organization published some guidelines that could help employers and employees better deal with the new reality of remote working. The authors of this report indicated that, “in such an unexpected and urgent situation as the COVID-19 pandemic, both employers and workers may be unprepared physically, mentally or infrastructure-wise to meet all challenges posed by working from home” (p. 13, [3]). This is so because the demands of remote work under lockdown circumstances are different. Remote workers had to wrestle with the staying-at-home of other members of the household and high levels of uncertainty about work itself, health, and the economy. The relationship between work, remote work in this case, and perceived work stress changed. The objective of this study is to explore precisely how this change affected work productivity, satisfaction, and compromise as well as work–life balance for remote workers under the COVID-19 pandemic in Latin American countries.

### 1.1. Remote Work and Stress

Before the pandemic, research about the relationship between remote work and stress has produced unconclusive results. On one hand, there are those that found that telework reduced work role stress [4,5,6,7], although the magnitude of the effect appears to be small. At the same time, the studies showed that reduction in the stress was mediated by an increase in job autonomy: the more autonomy the teleworker had, the lower the stress. On the other hand, there are those that found that telework is related to higher levels of stress [8], specifically by increasing work–life conflict [9] or affecting work–life balance [10].

However, working remotely during the pandemic differs from previous remote working arrangements in several ways: (a) it was not voluntary, neither for the employer nor for the employee; (b) it did not take into consideration individual traits or organizational culture—all tasks that could be performed remotely were to be performed that way regardless of the employee’s ability to cope with social isolation or of the employer’s culture; (c) it was intensive, in the sense that it did not allow for periods of not working remotely as all the work had to be done that way; and (d) it coincided with the staying-at-home of other members of the household. All these elements accentuate more the nuances of working remotely rather than the benefits, thus, we posit that, under these circumstances:

**Hypothesis** **1** **(H1).** *Remote work demands (RWD) are positively related to work stress (STR).*

### 1.2. Remote Work and Work–Life Balance, Work Productivity, Work Satisfaction, and Work Compromise 

Remote work has been hailed by employees and employers alike for allowing a better work–life balance. As a work arrangement, remote work permits employees to perform their duties and tasks away from the office, thus giving them more autonomy and control over where to work and how to combine work and personal life. In doing so, it helps improve work–life balance and labor inclusion [11,12,13,14]. Notwithstanding, the blurred lines that separate work and personal life present challenges to remote working in relation to work–life balance. Research shows that overworking is a frequent behavior in those working remotely, with the subsequent extension of working hours and poorer work–life balance [15,16,17]. Similarly, highly mobile teleworking [18] as well as inexperience in working remotely [5] cause work–family conflict, which negatively affects work–life balance. Finally, on the negative side, working from home may add to the amount of family responsibility assumed by the person working remotely [19], thereby increasing opportunities for a possible interference of the family realm into the work realm. During the first year of the pandemic, one of the consequences of lockdown was the staying-at-home of all members of the household, thus increasing the possibility of assuming more family responsibilities and affecting the balance between life and work. Therefore, we propose:

**Hypothesis** **2** **(H2).** *RWD are negatively related to work–life balance (WLB)*.

Increased productivity has been a powerful reason for introducing remote working as a work arrangement in organizations [20,21]. Research shows that being able to work from home allows employees to work at their most productive time [22], avoid interruptions or distractions by colleagues [23], and, as a result, increase their productivity [24]. However, that same productivity is negatively affected by teleworking when there is not an adequate place from which to work [24] or there are constant interruptions caused by children or adults that need assistance [25]. Since these last two characteristics are present in the actual health situation, we propose:

**Hypothesis** **3** **(H3).** *RWD are negatively related to work productivity (WP).*

Along the same lines, working remotely has been associated with higher job satisfaction, although, as a whole, the evidence is not consistent. On one hand, studies show a positive relation between telework and employees’ satisfaction with their work [5,26,27,28]. On the other hand, the evidence supporting higher job satisfaction for teleworkers is not robust and appears to be linked, according to [29], to the intensity of their work: teleworkers working more than 15.1 h a week rated the relationship poorly, while those working less showed a positive relationship between telework and job satisfaction. A reason for this decrease in work satisfaction is related to the increase in social isolation that remote workers may experience. Telework reduces interpersonal interactions and weakens links with other workers, thus causing workers to feel less connected and supported [30]. This need for relatedness is necessary for the experience of job satisfaction [27], thus, considering that, during the pandemic, telework became the working norm (more than 15.1 h a week) and was applied to most co-workers and supervisors, we propose the following:

**Hypothesis** **4** **(H4).** *RWD are negatively related to job satisfaction (WS).*

As it happens with the relationship between remote work and job satisfaction, the relation with job engagement is ambiguous at best. The positive effect found between one and the other is rather small [23,31]. Furthermore, in a quasi-experiment, Delanoeije and Verbruggen [32] found that the general level of work engagement did not change after employees were allowed to telework compared to their levels before when they were working at the office. On the negative side, Sardeshmukh et al. [7] found that telework is negatively related to job engagement and that job demands and resources mediate these relationships. Along the same lines run the results of other studies [33,34,35]. Thus, we posit the following:

**Hypothesis** **5** **(H5).** *RWD are negatively related to job engagement (WC).*

So far, we have discussed the direct relationship of remote work demands and stress, and remote work and work–life balance, productivity, job satisfaction and job engagement. However, as the literature also shows, there are mediator elements that work as channels through which remote working influences work–life balance, productivity, job satisfaction, and job engagement. For example, telework and job satisfaction is partially mediated by work–family conflict and co-worker relationships [5], and significantly mediated by decreased work–life conflict, as well as decreased information exchange frequency, stress from interruptions, and decreased involvement in office politics [28]. Golden [36] also found a partial mediation of leader–member exchange, team-member exchange, and work–family conflict in the curvilinear association between extent of telecommuting and job satisfaction. 

Similarly, Gajendran [5] found that perceived autonomy partially mediated the relationship between remote working and job performance, and Delanoeije [37] showed that the relation between remote work and work–family conflict was partially mediated by transitions from the work domain to the home domain on teleworking days. Furthermore, when looking at the relationship between remote work and job engagement, Gerards [38] identified a mediation of transformational leadership. 

The different mediations show that the relationships between remote working and the different outcomes are somewhat complex, and beg the possibility of identifying other mediations. The remote work taken place during the pandemic, as we have mentioned before, is characterized by different conditions from the previous remote work arrangements: (1) it was not voluntary; (2) it included almost everybody whose work can be performed remotely; (3) it was sudden; and (4) all members of the household were to remain at home carrying on their daily routines—work, school, etc. This scenario puts the now remote workers under stress, which originated in work demands that might affect or compromise their work performance, satisfaction, and work–life balance. Thus, we proposed that work stress could be working as a mediator for all the relationships between remote work and the different outcomes.

**Hypothesis** **6** **(H6).** *STR mediates the relationship between RWD and WLB.*

**Hypothesis** **7** **(H7).** *STR mediates the relationship between RWD and WP.*

**Hypothesis** **8** **(H8).** *STR mediates the relationship between RWD and WS.*

**Hypothesis** **9** **(H9).** *STR mediates the relationship between RWD and WC.*

### 1.3. Gender Differences

There is one more element that we would like to look at: gender. As Allen et al. [30] indicate, there is little evidence that gender influences the relationship between telework and the different outcomes. Specifically, Gajendran [5] found no evidence that gender played a role in the relationship between telework and job satisfaction, job performance, work–family conflict, or work stress. However, one disadvantage identified for those working from home is that the remote worker might be expected to shoulder in more home responsibilities. After all, they are “staying at home”. This rationale affects more women than men. As Hammer et al. [19] found, flexible work arrangements (location and timing flexibility) were positively reported in wives’ reports of family interferences with work one year later, but not in husbands’ reports. Similarly, in their research, Rodriguez-Mondroño [18] identified that men and women use their opportunities of flexible working in different ways, which leads to different outcomes for well-being, work–life balance, and work intensification. They also showed that more women than men who telework at home perceive job insecurity, which is recognized as a significant cause of stress [39]. In the circumstances of lockdown and general remote work due to the pandemic, plus the constant presence of children or adults, it is foreseeable that household responsibilities would fall under women’s duties who, in turn, would have to face double and triple burdens: taking care of the house, children’s care and education, and work at the same time and place. Thus, we propose that:

**Hypothesis** **10** **(H10).** *There will be a difference between men and women in their relationship between RWD and STR, WLB, WP, WS and, WC.*

**Figure 1:**
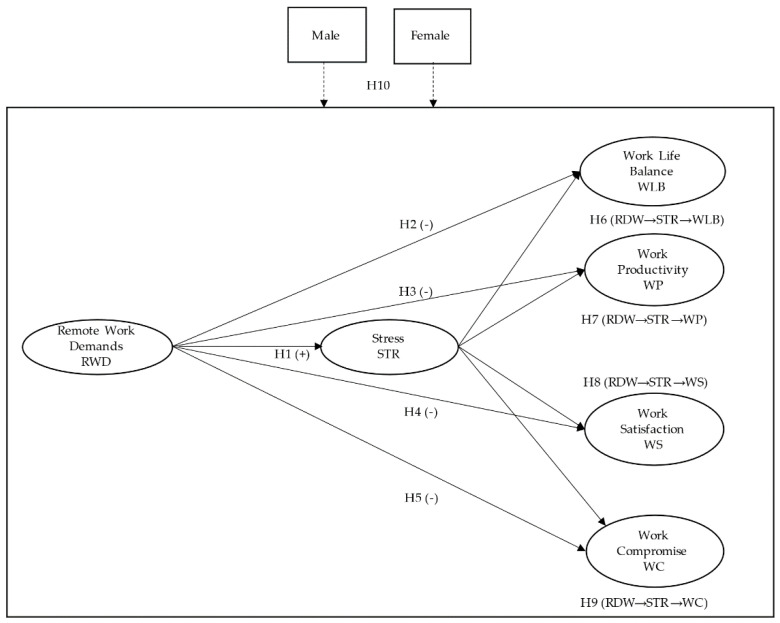
Conceptual Model.

The final conceptual model looks as Figure 1 shows:

## 2. Materials and Methods

### 2.1. Sample

The minimum sample size needed for testing the conceptual model was determined a priori through a statistical power analysis using G*Power 3.1.9.2. software. As recommended by Lakens et al. [40], we used a one-tailed test with an expected effect size of 0.05, significance level 0.05, an expected statistical power of 0.90, and two defined predictors, RWD and STR, that worked as exogenous variables and had a direct effect on the outcome variables. Results showed a recommended minimum size of 218. For H10 we used a two-tailed test with same input parameters. Minimum sample size recommended was 213.

We collected data during the first stage of the generalized lockdown that took place in South America: from 24 April to 25 May 2020. At this time, most countries in Latin America were experiencing strict lockdowns that limited circulation within the cities and countries, prohibited mass gatherings indoors and outdoors, and allowed only essential services and related activities to operate. We opted for a convenient sample and the questionnaire that we developed was disseminated through the professional networks of the researchers (i.e., LinkedIn). The only inclusion criteria were that the respondent was teleworking at the time. No exclusion criteria were used. Initially, we received 1874 responses. After discarding those that were incomplete, we ended up with a sample of 1285 respondents, which means 68.5% of effective participation. Most of the respondents were from Colombia (54.8%), 39.7% were from Ecuador, and 5.5% were from other countries of the region. The average age was 29.1 and 65.9% were female, while 34.1% were male. Of those, 68.6% were married and 49.3% lived with children. About a third of the sample had a college degree (33.8%) and 60.8% had a graduate education. Most of them worked in education (44.4%) and the second-best represented sector was service (18%). Finally, 89.5% of the respondents had been in their current job more than a year. 

### 2.2. Measures

To measure Remote Work Demands (RWD), we adapted three items from the Quantitative Workload Inventory [41]. Respondents had to compare actual remote work with previous on-site-jobs, and answer if they felt they had to work very fast, work very hard, or that they had a great deal to do. They could choose between three options: better, the same, and worse. 

To measure work stress (STR), we used five items from Folkman and Lazarus’s (1985) Work Stress Questionnaire [42] that measures the perceived change in the emotional state of a person as a consequence of facing difficult situations at work. The items were rated on a 7-point Likert scale where 1 is never and 7 is always. An example of the items used is: how frequently, since I have been working remotely, have I felt angry?

Finally, to measure work–life balance, work productivity, work satisfaction, and job engagement, we created four specific questions for this study. Respondents were asked to compare remote work and previous on-site-jobs and answer: (1) My work and life balance is…; (2) My work productivity has…; (3) My work satisfaction is…; (4) My job engagement is…, using better, the same, or worse as responses.

The Ethics Committees from our academic institutions approved the research (Act 140, 2020), and we developed an online questionnaire using QuestionPro^®^, Austin, TX, USA. Participation in the research was defined as voluntary, anonymous, confidential and without risks for the participants. Before completing the questionnaire, the participants signed an informed consent form.

### 2.3. Data Analysis

To analyze the data, we used partial least-squares-based structured equation modeling (PLS-SEM). This model consists of two elements: the measurement model and the structural model. The first one is defined as a reflective model because the items are considered a reflex of the constructs. In the second model, the RWD construct is used as an exogenous variable while the other constructs of the model are defined as endogenous variables. To analyze the data, we used SmartPLS3 [43].

Results from the PLS-SEM were evaluated for both models. For the measurement model we first estimated the reliability using the rho_A and Composite Reliability (CR) coefficients. Then, we calculated the convergent validity through external loads and the average variance extracted (AVE). As a last step we assessed the discriminant validity using the heterotrait–monotrait (HTMT) ratio of the correlations suggested by [44]. For the evaluation of the structural model, we used the explained variance effect size, predictive power, coefficient magnitude, and statistical significance for each of the paths proposed in the conceptual model. Finally, for the comparison between groups (male vs. female), we used the PLS-MGA approach [45], since it can determine whether there are significant differences among the estimated parameters for each of the groups (e.g., internal weights, external loads, path coefficients).

## 3. Results

### 3.1. Measurement Model Assessment

Reliability was evaluated using the internal consistency method. All the six variables showed adequate levels of reliability. The rho_A coefficient, as well as the composite reliability, reached values over 0.65 and lower than 0.95. The literature suggests this threshold is acceptable and satisfactory when assessing reliability (see Table 1).

Convergent validity indicates that a construct measures in a similar way to other constructs in the conceptual model. This criterion was applied to the constructs as well as to each of the indicators. As an estimator for the constructs, we used the average variance extracted (AVE). An acceptable AVE is 0.50 or higher, indicating that the construct explains at least 50 per cent of the variance of its items, thus an adequate level of validity [46]. For the indicators, we used two criteria: external loads (above 0.60) and the variance inflation factor (VIF). The VIF is often used to evaluate collinearity of the formative indicators. VIF values of 3.0 or lower are recommended for initial models in social sciences [46]. All indicators reached the values of reference, indicating that all constructs have convergent validity (see Table 1).

Discriminant validity is the extent to which a construct is empirically distinct from other constructs in the model. The process widely accepted to calculate the discriminant validity is the HTMT ratio, which measures similarity among latent variables [47]. The literature suggests a threshold value of 0.85, and that confidence intervals do not include 0 between the lower and upper limit (See Table 2). The results of both analyses meet the mentioned criteria which allows us to affirm that the constructs of the model have discriminant validity.

### 3.2. Structural Model Assessment

Results for the structural model assessment are presented in Table 3. RWD has a direct effect on STR: it is positive, statistically significant (*p* < 0.01), and explains 7% of the construct variance. Our H1 is supported, and we can assume that people working remotely perceive higher levels of stress as a consequence of the higher demands they face when working from home during the COVID-19 pandemic.

The direct effect of RWD on perceived WLB is negative and statistically significant (β = −0.225; *t* = 7.624; *p* < 0.01), supporting H2. Contrary to our hypothesis, the direct effect of RWD on WP is positive (β = 0.120; *t* = 3.817; *p* < 0.01), thus H3 is not supported. This effect is interesting and could be explained by arguing that perhaps, for remote workers, demands turn into challenges that push them to increase their efforts and meet them in the short-term. The direct effect of RWD on WS is negative (β = −0.190; *t* = 6.659; *p* < 0.01), lending support to H4. Conversely, the effect of RWD on job engagement (WC) is positive and significant (β = 171; *t* = 5.638; *p* < 0.01), leaving H5 without support. The same rationale used to explain results contradicting H3 can be used here: initial demands might have been assumed by remote workers during the pandemic as challenges that strengthen their compromise with their employer.

The size of the effect was assessed using Cohen’s [47] criteria: *f*^2^ > 0.02 is considered small; *f*^2^ > 0.15 is considered medium; and *f*^2^ > 0.35 is large. In this research, RWD effects on all five endogenous variables, although significant, are small (see Table 3).

Our conceptual model emphasizes a possible mediating effect of STR in the relationship between RWD and the endogenous variables: WLB, WP, WS, and WC. To identify this indirect effect that would explain the relationship, we used the approach suggested by Zhao [48]. This approach analyzes not only the significance of the relationship but also the direction of each of them (see Table 3). 

We found an indirect effect of RWD on WLB (*t* = 6.290 *p* < 0.01; CI −0.074, −0.039) and WS (*t* = 8.189 *p* < 0.01; CI −0.112, −0.069) through work stress (STR) that points in the same direction as the direct effect. These findings lend empirical support to H6 and H8 and identify a complementary partial mediating effect. In other words, the perceived stress while working remotely helps explain the reduction in levels of work–life balance and work satisfaction reported by the respondents.

We also found an indirect effect of RWD on WP (*t* = 5.721; *p* < 0.01; CI −0.074, −0.037) and WC (*t* = 5.898; *p* < 0.01; CI −0.080, −0.041) through work stress (STR) but they point in the opposite direction of the direct effect found previously. These results suggest a competitive partial mediating effect [48] and also support H7 and H9. The findings of a mediating effect of this characteristic indicate that remote work demands one obtains higher levels of productivity and engagement in the short-term during the pandemic situation, but, if the situation were to remain the same or increase in the middle-term, the worker would not be able to keep the levels of energy, effort, and compromise when the perception of work stress appears.

Finally, regarding H10, we did not find any significant differences in the relationships of the conceptual model between men and women (see Table 4).

Similarly, we did not find any significant differences in the indirect effects that would suggest a mediation of work stress upon the relationships between RDW and the outcome variables or groups. There was only one exception: we found a significant difference in the relation between STR and WP. The multigroup significance test showed significant values (β = 0.137; *p* < 0.01) when comparing original coefficients from the men’s group (β = −0.290; *t* = 5.874; *p* < 0.01) and the women’s group (β = −0.153; *t* = 4.278; *p* < 0.01). 

Lastly, we assessed the predictive PLS for all the indicators of the conceptual model, calculating the Q^2^ predict following the procedure suggested by Shmueli et al. [49]. When comparing the MAE (mean absolute error) value with the LM (lineal regression model) value for each indicator, we found major errors in two of the nine indicators analyzed: WP and WC. Therefore, we can only claim a medium predictive power for the model.

## 4. Discussion

We set out to identify the relationship between remote work demands, stress, and different outcomes during the COVID-19 pandemic in Latin America. Our results contribute to expand the scope of the relationship between remote work and stress, including elements such as work–life balance, productivity, work satisfaction, and job engagement. At the same time, we shed light upon the middle- and long-term effects of remote work. Working remotely during the pandemic was a positive way to keep work going, while also bringing positive economic effects. Contrary to the literature [24,25], remote work during the pandemic seems to have improved productivity and job engagement. 

However, our findings point to a downside to this improved productivity and engagement when the situation stretches in time, especially for those who bear high responsibilities, have to multitask, face round-the-clock availability, and have little or no time to rest. The positive effects on productivity and engagement are reduced when the stress they face due to these circumstances enters the equation. The competitive partial mediating effect [48] we found shows that stress lessens the positive effect of working remotely on productivity and engagement, and stress is an element that, under the actual circumstances, is increasing and would steadily affect those working outcomes. 

It is in this relationship between remote work and productivity that a difference between men and women appears. Our study shows that perceived stress affects productivity more acutely for men than for women. Although this is the only significant gender difference we found aligning our study with the argument that there is little evidence that gender influences the relationship between telework and the different outcomes [30], the relationship between stress and work productivity in men and women requires special attention and further research. 

Similarly, our findings expand the explanation of how stress impacts the relationship between working remotely and work–life balance, and between working remotely and job satisfaction. Stress acts as a complementary partial mediator, and, in doing so, it clarifies how remote work demands negatively affect the perception of work–life balance and job satisfaction. 

In line with these findings, organizations need to search for an equilibrium between the benefits of short-term remote work and the possible future impact they might have on the health and psychological well-being of their employees. In Latin America and the Caribbean, the use of teleworking services increased by 320% between the first and second quarters of 2020 [50], but the extent of this increase is not level with an increase in developing competencies to manage remote teams or manage one’s own remote work. Certain changes must be introduced at the operational and managerial levels to maintain steady productivity, and, at the same time, guard the well-being of the workers during these new circumstances. Asking for constant productivity and engagement from remote workers without considering the stress response to those demands is not sustainable and it will most likely end badly according to our findings.

At the same time, organizations should provide the psychological support their employees need using mitigation strategies such as telemedicine (psychology) and informal support groups [51]. Likewise, governments and businesses should develop policies that safeguard workers’ well-being—physical and mental—under the new circumstances.

Just as we looked into the mediating effect of perceived stress, future research needs to consider the role of organizational culture and leadership styles as mediators in the relationship between working remotely and the level of perceived stress. Similarly, other studies could look into the effect of variables such as age, gender, number of children, household members, characteristics of the house, among others, over the outcomes we have studied. For example, they could study the moderation effects of the number of children on the relationship between RWD and STR. Our research is not without limitations. The use of a convenient sample is a limitation that does not allow for generalizations beyond the results obtained with our sample. However, we consider that even though the use of PLS-SEM does not fix the selection bias, it strengthens the external validity of explorative studies due to the robustness of its analysis. The transversal design and the fact of collecting the data at the beginning of the pandemic is also a limitation. We suggest that future longitudinal studies could offer sufficient data to analyze the changes in the remote work dynamics at different times (one year, two years) and their effects on the levels of stress. It will also be helpful to use objective measures of productivity, satisfaction, work–life balance, and engagement, rather than a measure of perception of said issues. 

## 5. Conclusions

According to our data, for those who were able to change from traditional work during the pandemic in some countries in Latin America, remote work demands increased perceived stress, reduced work–life balance and work satisfaction, and increased productivity and engagement. Our explorative study also found that perceived stress has a competitive partial mediating effect that lessens the positive effect of working remotely on productivity and engagement. Conversely, perceived stress acts as a complementary partial mediator between remote work strengthening the negative impact of remote work demands and the perception of work–life balance and job satisfaction. The only significant gender difference we obtained was between working remotely and productivity: perceived stress affects men’s productivity more acutely than women’s productivity.

## Figures and Tables

**Table 1:** Measurement Model: Reliability and validity.

Variable	Outer Loading	VIF	Cronbach’s Alpha	Rho-A	CR	AVE
Remote Work Demands			0.682	0.896	0.904	0.518
RWD1	0.756 ***	1.347				
RWD2	0.740 ***	1.292				
RWD3	0.840 ***	1.343				
Stress			0.861	0.925	0.939	0.836
STR1	0.805 ***	2.188				
STR2	0.806 ***	1.899				
STR3	0.821 ***	2.061				
STR4	0.718 ***	1.590				
STR5	0.856 ***	2.604				
Work–Life Balance			1.00	1.00	1.00	1.00
Work Productivity			1.00	1.00	1.00	1.00
Work Satisfaction			1.00	1.00	1.00	1.00
Job Engagement			1.00	1.00	1.00	1.00

Note: *** *p* <0.001; VIF = variance inflation factor; Rho-A = Spearman’s correlation coefficient; CR = composite reliability; AVE = average variance extracted.

**Table 2:** Discriminant validity evaluation of the measurement model using HTMT.

Constructo	RWD	STR	WLB	WP	WS	WC
RWD	0.780					
STR	0.340 [0.271, 0.406]	0.802				
WLB	0.330 [0.264, 0.398]	0.285 [0.229, 0.339]	1.00			
WP	0.108 [0.067, 0.165]	0.185 [0.125, 0.245]	0.173 [0.121, 0.227]	1.00		
WS	0.326 [0.258, 0.395]	0.417 [0.364, 0.466]	0.356 [0.305, 0.411]	0.382 [0.330, 0.431]	1.00	
WC	0.147 [0.089, 0.217]	0.189 [0.127, 0.250]	0.088 [0.032, 0.143]	0.341 [0.285, 0.396]	0.306 [0.253, 0.356]	1.00

Note: On the diagonal, the square root of AVE. HTMT is shown above the diagonal; numbers in brackets represent the 95% bias-corrected and accelerated confidence intervals derived from bootstrapping with 5000 samples.

**Table 3:** Structural Model Assessment.

Hypothesis	Coef Path	*t*-Value	*p*-Value	95% CI	*f* ^2^	R^2^	Q^2^ Predict
H1 (RWD → STR)	0.269	10.258	0.000	[0.212, 0.317]	0.078	0.071	0.070
H2 (RWD → WLB)	−0.225	7.624	0.000	[−0.281, −0.166]	0.053	0.116	0.077
H3 (RWD → WP)	0.120	3.817	0.000	[0.058, 0.181]	0.014	0.041	0.003
H4 (RWD → WS)	−0.190	6.599	0.000	[−0.246, −0.132]	0.041	0.182	0.077
H5 (RWD → WC)	0.171	5.638	0.000	[0.112, 0.231]	0.029	0.056	0.011
H6 (RWD → STR → WLB)	−0.055	6.290	0.000	[−0.074, −0.039]			
H7 (RWD → STR → WP)	−0.054	5.721	0.000	[−0.074, −0.037]			
H8 (RWD → STR → WS)	−0.090	8.189	0.000	[−0.112, −0.069]			
H9 (RWD → STR → WC)	−0.059	5.898	0.000	[−0.080, −0.041]			

Note: 95% CI = confidence interval at 95% derived from bootstrapping for 5000. *f*^2^ = effect size; R^2^ = explained variance; Q^2^ predict = predictive effect.

**Table 4:** PLS-MGA Analysis.

Relationships	Coef Path Men	Coef Path Female	Coef Path Differences Male vs. Female	Value *p*-Original	Value *p*-New
RDW → STR	0.290	0.266	−0.025	0.674	0.652
RDW → WLB	−0.261	−0.217	0.045	0.234	0.469
RDW → WP	0.071	0.128	0.057	0.013	0.027
RDW → WS	−0.257	−0.160	0.097	0.056	0.112
RDW → WC	0.140	0.171	0.030	0.331	0.661

## Data Availability

Data will be available upon request to the corresponding author.
